# Transurethral laser lithotripsy using the Mitrofanoff urethral conduit for bladder stones: a case report

**DOI:** 10.1186/s13256-023-04131-5

**Published:** 2023-10-07

**Authors:** Shotaro Yamamoto, Takashi Kawahara, Tomoki Saito, Takahiro Hanai, Teppei Takeshima, Junichi Teranishi, Kazuhide Makiyama, Hiroji Uemura

**Affiliations:** 1 Departments of Urology and Renal Transplantation, Yokohama City University Medical Center, Yokohama, 2320024 Japan; 2 Department of Urology, Yokohama City University Graduate School of Medicine, Yokohama, 2360004 Japan

**Keywords:** Mitrofanoff conduit, Bladder stones, Cystolitholapaxy

## Abstract

**Background:**

The Mitrofanoff (appendicovesicostomy) procedure is a contraindicated urinary modification that maintains urinary continence by forming a flap-valve mechanism at the site of anastomosis between the appendage and bladder wall, which is used as a guide for urinary drainage. This technique has been used by intermittent self-catheterization patients who have difficulty voiding from the native urethra or in cases where voiding from the abdominal wall would improve quality of life. However, the risk of stone formation is high due to intermittent urinary catheterization using the Mitrofanoff conduit urethrostomy as a conduit.

**Case presentation:**

The patient was a 22-year-old Asian-Japanese woman. At 6 years of age, she underwent bilateral vesicoureteral reflux surgery, Mitrofanoff urethrostomy using the appendix, abdominal wall plication, and vaginoplasty using the ileum. During follow-up, ultrasound performed due to persistent pain during urinary drainage revealed a 26 mm bladder stone. We performed ureteroscopic lithotripsy 6Fr using ureteral access sheath and made lithotripsy using Ho: YAG laser, then successfully removed the target stone.

**Conclusions:**

We report a case of transurethral laser lithotripsy using the Mitrofanoff urethral conduit for bladder stones. Using with ureteral access sheath made lithotripsy and retrieved ureteral stone more effective.

## Introduction

The Mitrofanoff procedure is indicated for patients with bladder neck obstruction, urethral stricture, urethral injury, or congenital bladder dysfunction that prevents adequate voiding through the urethra [1]. A female patient with prune-belly syndrome (defined as the triad of dysplasia of the abdominal wall muscles, malformation of the renal urinary tract and—in males—a retained testicle) had lower urinary tract obstruction due to urethral dysplasia and a huge bladder, and was treated with the Mitrofanoff procedure [2]. In patients treated with this procedure, the bladder can only be used for urinary drainage using catheters, which increases the risk of urinary tract infections as well as the risk of bladder stones [3, 4]. The presence of serious medical illness precipitated by unhygienic food practices can worsen the prognosis of this serious condition [5, 6]. Open surgical bladder lithotripsy remains the preferred option in cases of large or multiple stones; however, less invasive percutaneous and transurethral (conduit) lithotripsy procedures are increasingly being reported ^3)^. In contrast, there are few reports on transurethral cystolithotripsy from a Mitrofanoff conduit. We herein report a case of ureteroscopic laser lithotripsy and retrieval using a ureteral access sheath for a patient with a Mitrofanoff conduit.

## Case presentation

**Fig. 1:**
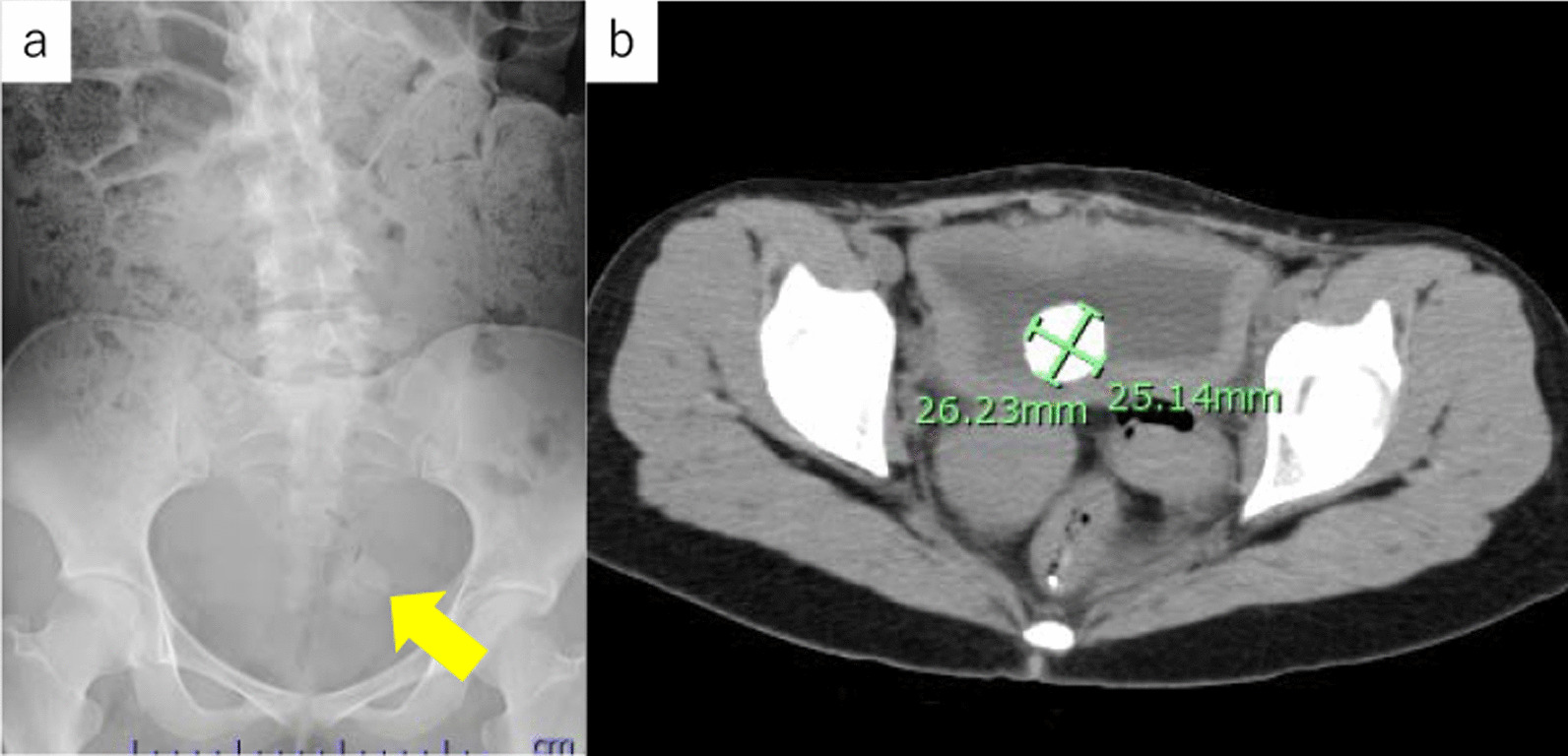
Preoperative **a** KUB and **b** CT (target stone: yellow arrow)

A 22-year-old Asian-Japanese woman was referred to our hospital for bladder pain. Computed tomography revealed 26 mm bladder stone. She had a huge bladder and lower urinary tract obstruction from the fetal stage. At birth, she was diagnosed with prune-belly syndrome (severe urethral stricture), cloaca, duplicated uterus, duplicated vaginal obstruction, bilateral VUR (vesicoureteral reflux), hypoplastic left lung, and right pneumothorax. She was treated by cystostomy with descending colostomy. At 6 years of age, she underwent bilateral vesicoureteral reflux surgery, Mitrofanoff urethrostomy using the appendix, abdominal wall plication, and vaginoplasty using the ileum. CIC (clean intermittent. catheterization) was performed 6 times a day and was successfully performed without urine leakage. During follow-up, ultrasound performed due to persistent pain during urinary drainage revealed a 26 mm bladder stone (Fig. 1). A stone was confirmed in the bladder by examination with a flexible cystoscope. The rigid cystoscope was inserted approximately 2 cm from the skin, but the patient experienced pain and the target stone could not be reached. One month later, she was admitted for transurethral laser lithotripsy using ureteroscopy with a ureteral access sheath.

Under general anesthesia, she was placed in the supine position and fixed with positional fixation equipment to facilitate easy intraoperative positioning. During surgery, we set her head up and rotated 15 degrees to the right for the easy handling of the ureteroscope easily and to keep the target from moving.

**Fig. 2:**
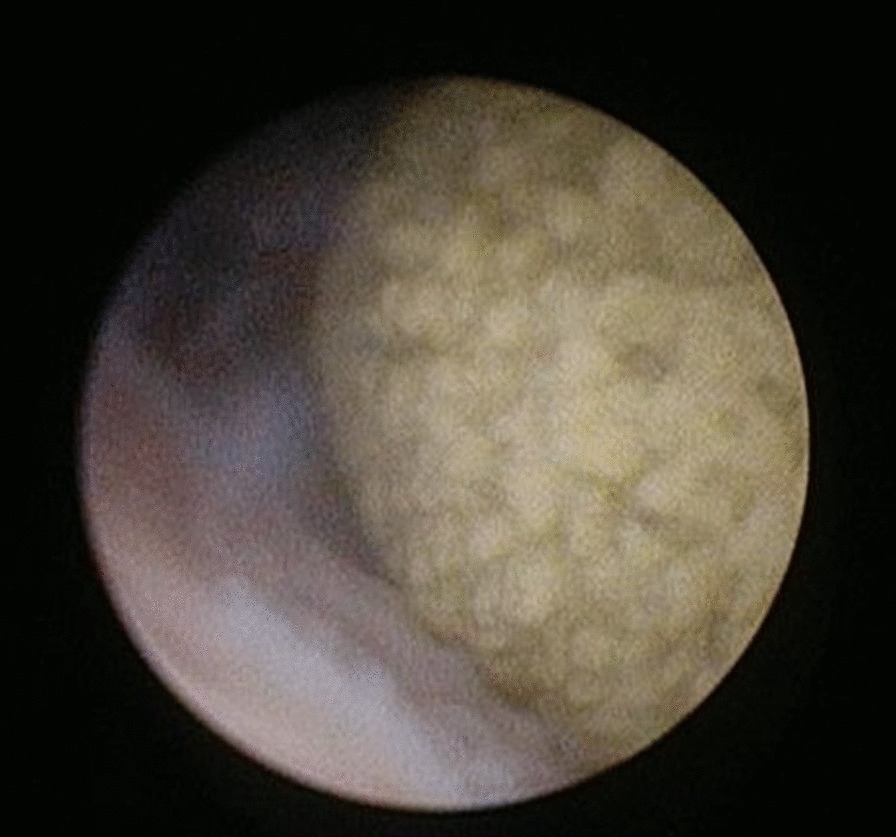
Intraoperative ureteroscopy

**Fig. 3:**
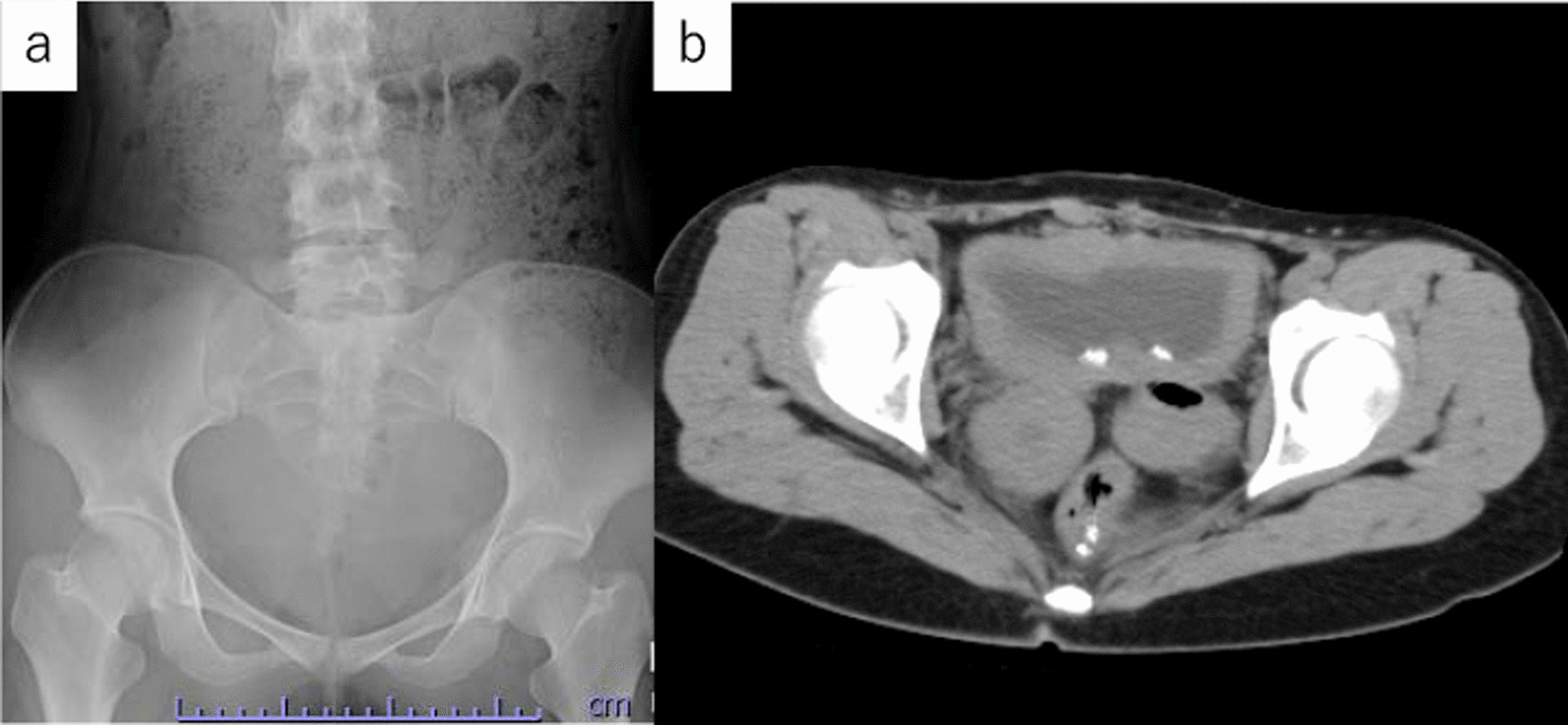
Postoperative **a** KUB and **b** CT

First, we tried to insert a 22Fr rigid cystoscope from the conduit, but the conduit was narrow, and we gave up inserting the cystoscope. A 6Fr rigid ureteroscope (Olympus, Tokyo, Japan) was smoothly inserted to reach the target stone. We found a yellow ball like formation stone attached to the bladder mucosa. Then, we inserted a ureteral access sheath (12/14Fr, COOK Medical, USA) under fluoroscopic guidance. Ho: YAG laser lithotripsy was performed using a 365 μm laser fiber with a laser setting of 1.0. We raised the laser setting to 2.0 J5 Hz due to the stiffness of the target stone (Fig. 2). Almost all fragment stones were removed using a basket device not to injure the urethral musoca, but some of the fragments were dusted. We concluded the operation after confirming that there were no large residual stones, and that only dusted small stones remained. KUB showed no residual stones, and CT showed tiny fragments at one month after surgery (Fig. 3). The stone chemical composition was 86% magnesium ammonium phosphate and 14% ammonium acid urate. She was free from pain and urinary continence showed sustained urinary continence.

## Discussion

Bladder calculi are a known complication of bladder augmentation that affect 12–52% of patients after enterocystoplasty. Risk factors include poor compliance with bladder washout, the need for CIC, the presence of foreign bodies, recurrent urinary tract infections, type of bowel segment used for augmentation, immobility, and bladder neck closure [3]. In strictest terms, bladder enlargement was not performed in this case, but in patients with a huge bladder and urethral obstruction associated with prune belly syndrome, bladder stones can easily form.

Various methods of cystolithotripsy have been reported, including open lithotripsy and percutaneous lithotripsy, but reports of cases with Mitrofanoff conduits are rare [3, 4, 7, 8]. There is concern about damage to the conduit during surgery; however, in the present case and previous reports, damage was prevented by the use of a ureteral access sheath over the conduit. Thomas *et al.* reported that 18–28 Fr ureteral access sheaths were used and that no patients had obvious conduit injuries that would have resulted in urinary incontinence [3]. The larger the sheath, the easier it is to expel stone debris and deliver and withdraw irrigation fluid; however, a larger sheath also increases the burden on the conduit. Accordingly, they advocated the use of an 18Fr sheath [3].

Sakly *et al.* first dilated the conduit with a 10–16 Fr navigation catheter and then a 15/16 Fr ureteral access sheath to care for conduit injuries [4]. The ureteral access sheath allows open and free drainage of irrigation fluid around the working instrument, which reduces both the intravesical pressure within the augmented bladder and the risk of rupture. Previous reports have shown that the surgical operation time is approximately 2.5–3 hour and that patients can leave the hospital within a few days after surgery [4, 7].

In the present case, the patient underwent surgery due to cystitis symptoms associated with an enlarged bladder stone. Although the patient’s symptoms improved after surgery and the diameter of each residual stone was less than 4 mm, stone recurrence was remained possible. In this case, we restricted the surgical operation time to less than 2 hour to avoid postoperative urinary tract infection. However, a longer surgical operation time would reduce the number of residual stones in cases in which good drainage of irrigation fluid can be achieved using a ureteral access sheath. Further study is needed to establish the ideal surgical management for complicated cases.

## Conclusions

We reported a case of transurethral laser lithotripsy using the Mitrofanoff urethral conduit for a patient with bladder stones. Using with ureteral access sheath made lithotripsy and retrieved bladder stone more effective. But careful management not to injure urethral mucosa and less operation time less than two hour are needed.

## Data Availability

Not applicable.
